# Comparison of Retzius-sparing versus anterior robotic-assisted radical prostatectomy in patients with prior transurethral resection of the prostate (TURP)

**DOI:** 10.1007/s00345-025-06112-3

**Published:** 2025-12-05

**Authors:** Viktoria Schütz, Gencay Hatiboglu, David Würkner, Mete Tekesin, Manuel Feisst, Stefan Duensing, Johannes Huber, Markus Hohenfellner, Basil Kaufmann

**Affiliations:** 1 Department of Urology, University Hospital Heidelberg, Im Neuenheimer Feld 420, 69120 Heidelberg, Germany; 2 Department of Urology, SLK Kliniken, Am Gesundbrunnen 20-26, 74078 Heilbronn, Germany; 3 Institute of Medical Biometry, University Hospital Heidelberg, Im Neuenheimer Feld 130.3, 69120 Heidelberg, Germany; 4 Molecular Urooncology, Department of Urology, University Hospital Heidelberg, Im Neuenheimer Feld 517, 69120 Heidelberg, Germany; 5 Department of Urology, University Hospital Zurich, University of Zurich, Frauenklinikstraße 10, Zurich, 8091 Switzerland

**Keywords:** Radical prostatectomy, Prostate cancer, Retzius-sparing radical prostatectomy, TURP, Functional outcome, Continence

## Abstract

**Background/objectives:**

Radical prostatectomy (RP) following previous transurethral resection of the prostate (TURP) is technically challenging due to an altered anatomy and fibrosis, often resulting in impaired functional outcomes. Open and anterior robotic approaches (aRARP) were evaluated in this setting, while data on Retzius-sparing robotic radical prostatectomy (rsRARP) is lacking. We aim to compare urinary continence, complications, and oncologic control between rsRARP and aRARP in patients with prior TURP.

**Patients and methods:**

A total of 65 patients with localized prostate cancer undergoing rsRARP or aRARP between 2010 and 2022 following prior TURP were analyzed. The primary endpoint was urinary continence recovery (max. one safety pad per day), immediately after catheter removal and at 12 and 24 months. Secondary endpoints included peri- and postoperative complications and biochemical recurrence (BCR)-free survival.

**Results:**

Of the included patients, 30 patients (46%) and 35 patients (54%) underwent rsRARP and aRARP, respectively. Median time between TURP and RP was 65 months (IQR 19–132). Immediate continence rate was 73.3% (*n* = 22) in rsRARP patients vs. 57.1% (*n* = 20) in aRARP patients. At 12 months, continence rates were 86.2% (rsRARP) vs. 91.2% (aRARP), and at 24 months, 80.8% vs. 93.9%, respectively. The surgical technique was not significantly associated with 12-month continence recovery, while nerve-sparing showed a trend towards improved outcomes. Complications occurred in 15.4% (*n* = 10), the highest being a grade IIIb according to the Clavien-Dindo classification. At 24 months, BCR-free survival was 89% (95% CI: 79–100) for rsRARP and 97% (92–100) for aRARP (*p* = 0.42).

**Conclusion:**

In patients with prior TURP, RARP using a Retzius-sparing approach is feasible and safe. While long-term continence outcomes are comparable to aRARP, rsRARP may offer improved immediate continence recovery, likely due to superior preservation of anatomical structures relevant to urinary function. These findings support the use of rsRARP even in surgically challenging post-TURP anatomy.

**Supplementary Information:**

The online version contains supplementary material available at 10.1007/s00345-025-06112-3.

## Introduction

Radical prostatectomy (RP) remains is standard surgical treatment for localized prostate cancer (PCa), offering excellent oncological outcomes in appropriately selected patients [1]. However, RP is associated with morbidity, particularly urinary incontinence and erectile dysfunction, which can significantly impact quality of life [2]. Over recent decades, surgical techniques have evolved from the open retropubic prostatectomy to minimally invasive laparoscopic and robotic-assisted techniques. These innovations have significantly contributed to improvements in perioperative morbidity and functional recovery [3–5].

Robotic-assisted radical prostatectomy (RARP) is now the predominant surgical approach, with the anterior transperitoneal technique (aRARP) being most frequently employed [6]. In 2010, Galfano et al. introduced the Retzius-sparing RARP (rsRARP), a novel approach leaving the Retzius space intact, thereby preserving key anatomical structures including the puboprostatic ligaments, endopelvic fascia, and the dorsal venous complex [7]. RsRARP gained significant attention for leaving critical anatomical structures essential for postoperative continence untouched e.g., pubourethral ligaments or the endopelvic fascia [8]. Therefore, rsRARP has been recognized due to its association with improved early continence recovery without compromising surgical safety [9]. However, some studies suggest that the limited anterior exposure may increase the risk of positive surgical margins (PSM), especially in anteriorly located tumors [6].

Performing RP in patients with prior TURP for benign prostatic hyperplasia (BPH) is surgically challenging. The altered prostatic and peri-prostatic anatomy resulting from TURP, including fibrosis, scarring, and loss of key anatomical landmarks, can complicate dissection and increase the risk of injury to vital structures (e.g. bladder neck or external sphincter) [10–12]. These anatomical changes may negatively affect functional outcomes (particularly urinary continence) and oncological control. For instance, a previously resected bladder neck often necessitates reconstruction and increases the risk of inadvertent ureteral injury [10, 11]. The risk of PSM and impaired anastomotic healing is also higher in this subgroup, necessitating a careful and tailored surgical strategy.

Despite the technical demands both aRARP and rsRARP have been employed in patients post TURP. However, data comparing both techniques in this context are limited. Most available evidence evaluates open or anterior robotic techniques, while the application and potential advantages of rsRARP in this subgroup remain underreported.

The aim of this study was to compare perioperative, functional, and oncological outcomes of rsRARP versus aRARP in patients with a history of TURP. Since patients after TURP represent a surgically challenging subgroup, a direct comparison of both robotic techniques may help to clarify whether one approach offers advantages regarding continence recovery, complications, or cancer control.

## Patients and methods

### Study design and population

We performed a retrospective analysis of a prospectively maintained database including 65 consecutive patients undergoing RARP between 2010 and 2022 for localized prostate cancer with a history of TURP for BPH. Patients with metastatic disease, preoperative urinary incontinence, or bladder disfunction were excluded. All patients underwent either rsRARP or aRARP. The study protocol was approved by the local ethics committee by the Medical Faculty of the University of Heidelberg (S-469/2023).

### Collected parameters

The following variables were collected: patient age at time of surgery, body mass index (BMI), prostate-specific antigen (PSA) at biopsy, time between TURP and RP, prostate volume assessed on preoperative multi-parametric MRI or trans-rectal ultrasound, preoperative pelvic floor muscle training, surgical approach, operating time, blood loss, bladder neck reconstruction, nerve-sparing, time to bladder catheter removal, ISUP grade group at biopsy and final histopathology after RP, pathological T and N stage (pT stage; ≤pT2, ≥pT3), surgical margins and their location, peri- and postoperative complications according to the Clavien-Dindo Classification [13].

### Surgical approach

RARP was performed either by the anterior approach as described by Guillonneau et al. [14], or the Retzius-sparing approach [7] using the da Vinci Si system (Intuitive Surgical, Sunnyvale, CA, USA). For the Retzius-sparing approach, dissection was performed transperitoneally through the rectovesical pouch. The seminal vesicles and vas deferens were identified and mobilized, followed by intrafascial posterior dissection of the prostate. The retropubic (Retzius) space and endopelvic fascia were not entered, and the puboprostatic ligaments and dorsal venous complex were left intact. The prostate was shelled out beneath the detrusor apron, and the vesicourethral anastomosis was performed posteriorly with barbed sutures, preserving anterior support structures that are associated with early continence recovery.

For the anterior approach, the peritoneum was incised anterior to the bladder to enter the Retzius space. The endopelvic fascia was opened bilaterally, the puboprostatic ligaments and dorsal venous complex were controlled and divided, and the bladder neck was dissected. Prostate dissection was performed in an antegrade fashion, followed by anterior vesicourethral anastomosis using the Van Velthoven running suture technique [24].

Pre- or intraoperative ureteral stenting was not routinely used. Stents were placed in rsRARP selectively in cases with impaired visualization of the ureteral orifices or when a heavily scarred bladder neck was anticipated to facilitate safe identification during dissection and anastomosis.

Nerve-sparing was applied based on the patients’ preoperative erectile function, personal preferences, and tumor grade. In both techniques, watertightness of the anastomosis was tested intraoperatively after completion. A periurethral suspension stitch as described by Rocco et al. [15] was done in both techniques. A 20 French Foley catheter was placed. Prior to catheter removal, cystography was performed. If extravasation was present, the catheter was not removed, and follow-up cystography was scheduled according to the severity of extravasation.

### Follow-up

A thorough follow-up was conducted. All patients received their first follow-up after catheter removal and immediate continence was noted. This was done via patient interview or questionnaire. Pad use per day was evaluated. Subsequent follow-up data collection was done every three months. Follow-up data was thoroughly examined and entered into the departments database.

### Study endpoints

The primary endpoint was the postoperative urinary continence recovery (UCR), which included patients who experienced no involuntary urine loss, as well as those with use of max. one safety pad per day, which allows for occasional spotting of urine during activities such as coughing, sneezing, or other strenuous actions [16]. Urinary continence was not considered achieved if patients used more than one pad per day or experienced grade II incontinence, characterized by frequent urine loss during normal activities (e.g. walking). Continence was evaluated immediately after catheter removal, and at 3, 6, 9, 12, 18, and 24 months post-surgery. For the purposes of this report, we present our data at immediate, 12, and 24 months post-surgery.

The secondary endpoints included oncological outcomes and peri- and post-operative complications. Oncological outcomes included the rates and location of PSM and BCR free survival. PSM was defined as the presence of tumor cells at the inked surface of the resected specimen, and BCR was identified by a postoperative PSA level of ≥ 0.2 ng/mL ^1^. Additionally, peri- and post-operative complications up to 90 days postoperatively were assessed and classified according to the Clavien-Dindo classification [17].

### Statistical analysis

Results are presented using descriptive statistics. Categorical variables are shown as frequencies and percentages, while continuous variables are expressed as medians and interquartile ranges (IQR). To compare categorical values, Fisher’s exact test and the chi-squared test were employed. The Mann-Whitney U test was used for comparing distributions. BCR free survival was estimated using Kaplan–Meier method and reported with 95% confidence intervals. Univariate logistic regression was performed to assess factors associated with UCR at 12 months. Variables for the multivariate logistic regression were selected based on clinical relevance, and univariate p-values ≤ 0.05. Variables showing statistically significant baseline differences between the surgical groups (Rocco stitch, pathological N-stage) were included to account for potential confounding. To reduce the risk of overfitting given the limited number of outcome events, the final model was restricted to five predictors: surgical technique, Rocco stitch, pathological N-stage, nerve-sparing status, and age at surgery. Complete case analysis was applied. We additionally fitted a Firth-penalized logistic regression as sensitivity analysis. All statistical tests were two-sided and a p-value < 0.05 was considered statistically significant in a descriptive sense, acknowledging the exploratory nature and the limited sample size. Statistical analyses were performed using R version 4.3.1 (R Foundation for Statistical Computing, Vienna, Austria).

## Results

### Baseline characteristics

Sixty-five patients were included in this evaluation (median age at surgery; 70 years, QR 66–76). The median time from TURP to surgery was 65 months (IQR 19.0–132.0). Thirty-two (49.2%) had stage ≤ pT2 and 33 (50.8%) patients had stage ≥ pT3 disease. Median PSA level was 6.80 ng/mL (IQR 4.07–11.1). Median time until catheter removal post-surgery was 21 days (IQR 14–22). Preoperatively all patients were fully continent. Baseline characteristics are shown in Table 1. Thirty patients (46.2%) underwent rsRARP and 35 patients (53.8%) underwent aRARP. Statistically significant differences among the groups were noted only for performed lymph node dissection (*p* = 0.030) and the Rocco stitch (*p* = 0.047; Table 1).

**Table 1:** Baseline characteristics of the cohort overall and by surgery technique

Parameter	Overall (n = *65*)	rsRARP (*n* = 30)	aRARP (*n* = 35)	*p* value
**Median age**, **yr (IQR)**	70 (66–76)	72 (66–76)	69 (66–75)	0.716
**Median PSA level**, **ng/mL (IQR)**	6.80 (4.07–11.1)	6.95 (4.80–12.3)	6.32 (3.65–11.0)	0.764
**Median BMI**, **kg/mq (IQR)**	26.0 (24.0–29.0)	25.0 (23.0–27.0)	27.0 (24.0–29.5)	0.314
**Median time from TURP to RP**, **months (IQR)**	65 (19.0–132)	76.5 (25.5–138)	64.0 (16.5–132)	0.734
**Median prostate volume before RP**, **mL (IQR)**	25.0 (20.0–30.0)	25.0 (21.3–30.8)	25.0 (19.0–30.0)	0.997
**Median operating time**, **minutes (IQR)**	227 (198–256)	222 (201–269)	227 (197–253)	0.896
**Median estimated blood loss**, **mL (IQR)**	400 (225–500)	300 (200–400)	500 (300–700)	0.674
**Nerve sparing**, **n (%)**	32 (49.2)	14 (46.7)	18 (51.4)	0.929
**Rocco stitch**, **n (%)**	53 (84.1)	33 (94.3)	20 (71.4)	**< 0.047**
**Preoperative pelvic floor training**, **n (%)**	9 (13.8)	4 (14.3)	5 (14.3)	0.994
**Tightness of anastomosis (intraoperative)**, **n (%)**	55 (87.3)	24 (82.8)	31 (91.2)	0.606
**Bladder neck reconstruction**, **n (%)**	24 (36.9)	10 (33.3)	14 (40.0)	0.857
**Median time to bladder catheter removal**, **days (IQR)**	21.0 (14.0–22.0)	21.5 (14.0–23.5)	21.0 (13.3–22.0)	0.659
**ISUP grade at prostate biopsy**				
1	11 (16.9)	5 (16.7)	6 (17.1)	0.945
2	31 (47.7)	13 (43.3)	18 (51.4)	
3	10 (15.4)	5 (16.7)	5 (14.3)	
4	5 (7.7)	4 (13.3)	1 (2.9)	
5	8 (12.3)	3 (10.0)	5 (14.3)	
**ISUP grade at RP**				
1	3 (4.6)	1 (3.3)	2 (5.7)	0.987
2	34 (52.3)	14 (46.7)	20 (57.1)	
3	21 (32.3)	12 (40.0)	9 (25.7)	
4	3 (4.6)	1 (3.3)	2 (5.7)	
5	4 (6.2)	2 (6.7)	2 (5.7)	
**Pathological T stage**, **n (%)**				
≤ pT2	32 (49.2)	13 (43.3)	19 (54.3)	0.679
≥ pT3	33 (50.8)	17 (56.7)	16 (45.7)	
**Pathological N stage**, **n (%)**				
pN0	51 (78.5)	19 (63.3)	32 (91.4)	**0.030**
pN1	3 (4.6)	1 (3.3)	2 (5.7)	
pNX	11 (16.9)	10 (33.3)	1 (2.9)	
**Surgical margin status and localization**, **n (%)**				
Negative	48 (43.1)	19 (63.3)	19 (54.3)	0.945
Positive	27 (41.5)	11 (36.7)	16 (45.7)	
Bladder neck	3 (10.7)	1 (3.3)	2 (5.7)	
Apex	10 (35.7)	5 (16.7)	5 (14.3)	
Lateral	3 (10.7)	2 (6.7)	1 (2.9)	
Cranial	6 (21.4)	1 (3.3)	5 (14.3)	
Multifocal	6 (21.4)	3 (10.0)	3 (8.6)	
**T-stage and positive surgical margin**, **n (%)**	27 (41.5)	11 (36.7)	16 (45.7)	
≤ pT2		3 (10)	5 (27.8)	
≥ pT3		8 (26.6)	11 (68.8)	
**BCR free survival at 24-months**, **% (95% CI)**	93 (87–99)	89 (79–100)	97 (92–100)	0.42

*rsRARP* retzius sparing robotic assisted radical prostatectomy, *aRARP* conventional robotic assisted radical prostatectomy, *BMI* body mass index, *IQR* interquartile range, *RP* radical prostatectomy, *TURP* transurethral resection of the prostate

### Urinary continence recovery

Urinary continence recovery rates are summarized in Table 2. Overall, immediate urinary continence was achieved by 65% (73.3% in rsRARP vs. 57.1% in aRARP; *p* = 0.473). At 12-month, the overall UCR rate was 82.6%. It was higher in the aRARP group with 91.2% (31/35) compared to 86.2% (*n* = 25) in rsRARP group (*p* = 0.777). By 24-months, 80.0% (*n* = 52) patients reported of urinary continence, with similar rates in the rsRARP (80.8%) and the aRARP group (93.9%), without statistically significant differences (*p* = 0.631).

**Table 2:** Urinary continence recovery (UCR) overall and by surgical technique

Parameter	Overall (*n* = 65)	rsRARP (*n* = 30)	aRARP (*n* = 35)	*p*-value
**Immediate UCR**, **n (%)**	42 (64.6)	22 (73.3)	20 (57.1)	0.473
Missing	0 (0)	0 (0)	0 (0)	
**12-month UCR**, **n (%)**	56 (82.6)	25 (86.2)	31 (91.2)	0.777
Missing	2 (3.1)	1 (3.3)	1 (2.9)	
**24-month UCR**, **n (%)**	52 (80.0)	21 (80.8)	31 (93.9)	0.631
Missing	6 (9.2)	4 (13.3)	2 (5.7)	

UCR (Urinary continence recovery) = up to one pad per day; r*rsRARP* retzius sparing robotic assisted radical prostatectomy, *aRARP* conventional robotic assisted radical prostatectomy, *BMI* body mass index, *IQR* interquartile range

**Fig. 1:**
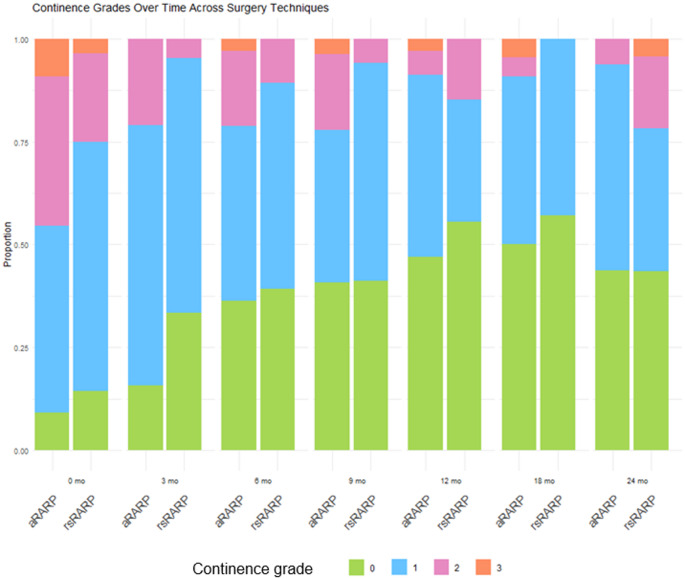
Continence recovery by grade over time and across the two surgery techniques

Figure 1 shows the distribution of incontinence grades (0-III) over time and for both surgical techniques. The rate of UCR increased steadily over time for both groups. Severe incontinence (Grade III) was relatively rare across both groups and time points. The highest incidence of Grade III incontinence immediately post-surgery was observed in 9.1% in the aRARP group compared to only 3.6% in the rsRARP group. However, by 24 months, severe incontinence was almost nonexistent. Uni- and multivariate logistic regression analyses for UCR at 12 months are summarized in Table 3. In the univariate analysis, only nerve-sparing significantly improved continence (OR: 17.3, 95% CI: 1.94–2280, *p* = 0.006). In the multivariate analysis, no variable was significantly associated with UCR.

**Table 3:** Univariable and multivariable logistic regression analyses testing UCR 12 months after RP

Variables	UCR at 12 months after surgery					
	Univariate Model (Firth)			Multivariate Model (Firth)		
	OR	95% CI	*p*-value	OR	95% CI	*p*-value
**Age at surgery**	1.00	0.90, 1.14	> 0.9	1.08	0.92, 1.29	0.4
**BMI**	1.05	0.83, 1.30	0.7	–	–	–
**Pathological T stage**						
Organ confined (≤ pT2)	Ref.					
Non organ confined (≥ pT3)	2.36	0.52, 14.0	0.3	–	–	–
**Pathological N stage**						
pN0	Ref.					
pN1	4.02	0.33, 35.6	0.2	0.51	0.00, 9.12	0.7
pNx	0.29	0.00, 2.79	0.3	0.33	0.00, 5.54	0.5
**Prostate volume**	1.01	0.94, 1.06	0.9	–	–	–
**Nerve sparing (vs. no)**	17.3	1.94, 2280	**0.006**	8.28	0.86, 1079	0.071
**Surgical technique**						
rsRARP	Ref.					
aRARP	0.63	0.13, 2.83	0.5	0.75	0.12, 0.490	0.8
**Pelvic floor training**,** preoperative ** **(vs. no)**	0.76	013, 7.95	0.8	–	–	–
**Bladder neck reconstruction (vs. no)**	2.83	0.54, 28.4	0.2	–	–	–
**Rocco stitch (vs. no)**	1.33	0.13, 7.87	0.8	0.86	0.06, 7.09	0.9

UCR (Urinary continence recovery): Incontinence grade 0–1 or max. 1 safety pad per day, *TURP* previous trans-urethral resection of the prostate, *RP* radical prostatectomy, *aRARP* anterior robot-assisted radical prostatectomy, *rsRARP* Retzius-sparing robot-assisted radical prostatectomy, *BMI* body mass index<, *OR* Odds Ratio, *CI* Confidence Interval

### Oncological outcome

Final histology revealed advanced disease (≥ pT3) in 50.8% (*n* = 33) of patients (Table 1) with no statistically significant difference between the two groups (*p* = 0.679). The rate of PSM was 45.7% in the aRARP group vs. 36.7% in the rsRARP group (*p* = 0.945). An ISUP grade group of ≥ 3 was observed in 50% in the rsRARP and 37.1% in the aRARP group. Positive lymph nodes (N1) were observed only in 3.3% and 5.7% of patients in the rsRARP and aRARP group, respectively.

**Fig. 2:**
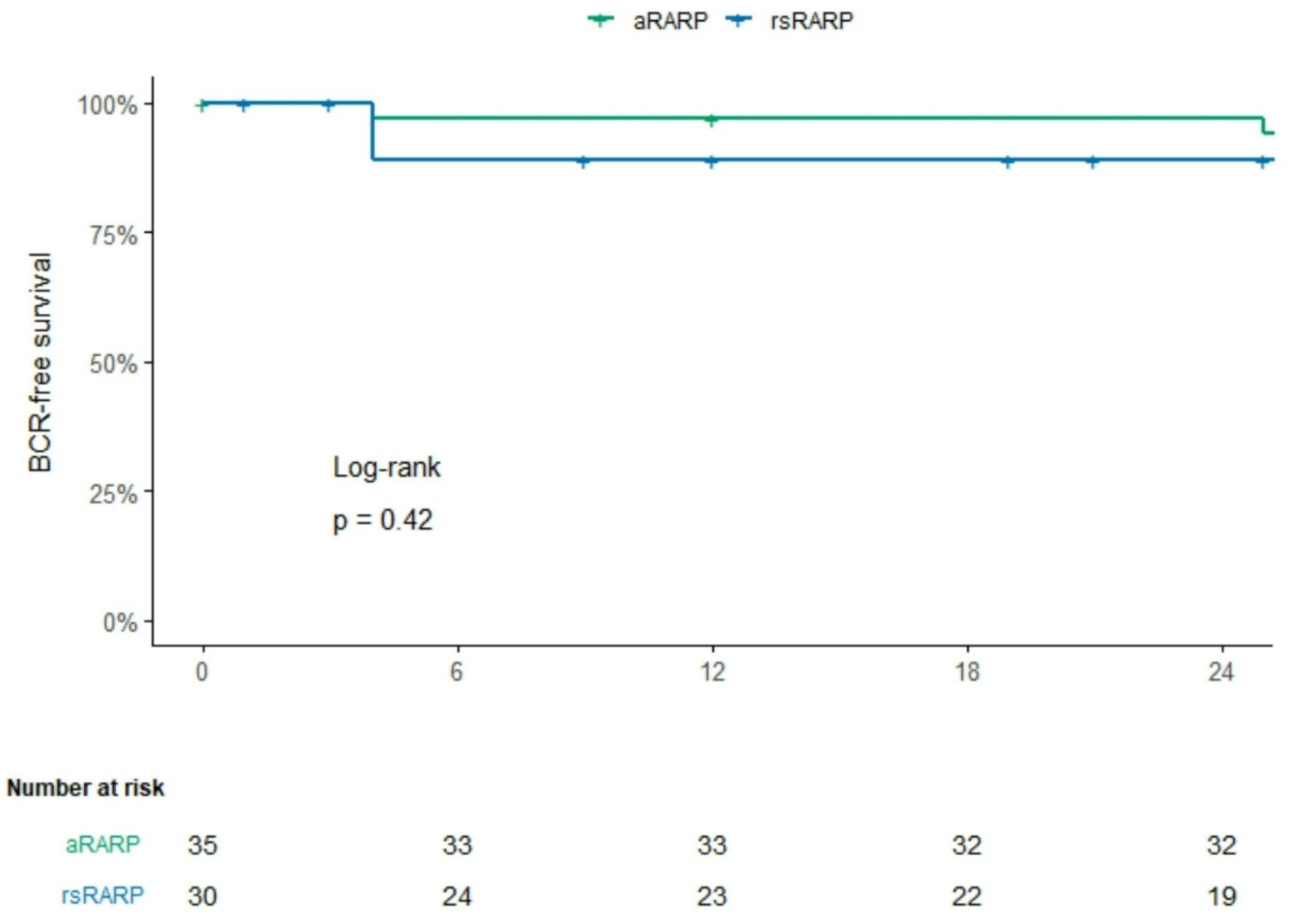
Kaplan-Meier curves with BCR-free survival according to surgical approach

Kaplan-Meier analysis curves for BCR-free survival are shown in Fig. 2. BCR-free survival rates for the aRARP group was consistent at 97.1% (95% CI: 91.5–100%) during follow-up. In the rsRARP group the BCR-free survival rate was 88.9% (95% CI: 77.8–100%) throughout the study period.

### Complications

Peri- and post-operative complications occurred in 12.3% (*n* = 8) of cases. Two patients (5.8%) in the aRARP group and six patients (20%) in the rsRARP group experienced complications. Main complications included lymphoceles in 4.6% (*n* = 3) and wound healing disorders in 3.1% (*n* = 2). An intra-operative ureteral injury only occurred in one patient (1.5%, rsRARP group). One patient after rsRARP (1.5%) experienced temporary hip flexor weakness due to compression of the femoral nerve by a hematoma. One vesicorectal fistula was observed after rsRARP. Complications according to the Clavien-Dindo Classification [13] are presented in Supplementary Table 1.

## Discussion

In this single-center study, we evaluated UCR and oncologic outcomes following RARP in men after TURP, comparing rsRARP and aRARP. Both techniques proofed to be feasible and effective in this surgically challenging patient population. Given that postoperative continence is the functional domain most affected by TURP-related anatomical disruption, urinary continence recovery was selected as the primary functional endpoint of this study. Immediate continence favored rsRARP (73.3% vs. 57.1%), while long-term continence recovery at 12 and 24 months was comparable. These results align with previous studies in TURP-naïve patients, which consistently show faster continence recovery after rsRARP [18]. One report on rsRARP after TURP showed inferior continence outcomes in patients with prior TURP compared to TURP-naïve patients with lower immediate and 12-month continence rates (68% vs. 94%) [19]. In our cohort 12-months continence rates are superior to these results.

The overall inferior UCR in post-TURP patients is likely attributable to anatomic disruptions, especially fibrosis, loss of bladder neck integrity, and a shorter functional urethral length which negatively influence functional preservation during dissection. Several studies have underscored the detrimental impact of TURP on sphincter mechanism and anastomotic healing [11, 20–22]. Loss of the bladder neck and internal sphincter deficiency may explain delayed or incomplete continence recovery in this subgroup. Su et al. found that patients undergoing RARP after TURP reported worse urinary symptoms compared to TURP-naïve patients. The study indicated poorer early continence rates in patients after TURP (43.7% vs. 68.6% at 3 months), though differences leveled out by the 12-month follow-up [23]. Others reported similar 12-month continence rates between TURP and TURP-naïve groups [24]. However, UCR is generally regarded poorer in patients after TURP across several follow-up periods [25]. Time since TURP appears to influence outcomes. A delay of over 60 months between TURP and RARP is regarded beneficial for UCR [12], possibly due to partial resolution of inflammation and tissue remodeling. In our cohort, the median interval between TURP and RP was 65 months.

Over half of patients had ≥ pT3 disease on final pathology, with an overall PSM rate of 41.5%. The PSM rate was lower in the rsRARP group (36.7%) despite a higher proportion of patients with ≥ pT3 disease. This observation challenges frequent concerns that rsRARP may compromise oncological safety due to limited anterior exposure [6]. Several studies have shown increased PSM rates after TURP and a worse oncological outcome [25]. These results have been attributed to anatomical changes complicating dissection [11]. Our results are further supported by previous reports showing higher PSM rates in patients after TURP [26]. However, the prognostic impact of PSM on long-term oncological outcome is still unclear and is likely context-dependent [27]. Compared to previously published data the PSM rate in our cohort was higher most likely due to a large proportion of stage ≥ pT3 disease, a long recruiting period and changes in pathology reporting standards. However, as the PSM rate was not the main focus of this evaluation no standardized, centralized pathological review was done.

Despite the PSM-rate, BCR-free survival at 24 months was favorable in both groups (93% overall) with no statistically significant difference. Regarding BCR-free survival our results are superior compared to previous evaluations, in which a history of TURP significantly increased the risk of BCR compared to patients without previous TURP [28]. However, long-term follow-up is needed.

Overall complication rates were low, with few Clavien-Dindo grade ≥ IIIb events (e.g., symptomatic lymphoceles (2%), ureteral injury (1%), vesicorectal fistula (1%)), which is in line with previous evaluations [19]. Ureteral injury occurred only in one patient, which is rare both in rsRARP and aRARP [29]. The vesicorectal fistula also occurred in a patient after rsRARP. Vesicorectal or other urorectal fistula are also very rare (< 1%), but serious complications after RARP and usually originate in the bladder neck [30]. Other studies reported higher complication rates compared to RARP without previous TURP, longer operative times and a higher blood loss due to increased intraoperative difficult [11, 25].

Limitations to this study include the retrospective nature and single-center design with possible selection bias and limited generalizability. The small sample size reduces statistical power, therefore, clinically relevant effects may not have reached statistical significance, and non-significant results should not be interpreted as evidence of no effect. As this was a retrospective exploratory study of a rare patient population, no a priori sample size calculation was feasible. The absence of a TURP-naïve control group precludes direct comparisons with standard RARP outcomes. Additionally, missing follow-up data and reliance on patient-reported UCR and pad use per day (without pad weight testing) are further limitations.

## Conclusions

Our findings support the safety and feasibility of both aRARP and rsRARP in patients with prior TURP. RsRARP may offer an advantage in early continence recovery while maintaining comparable oncologic outcomes. Given the anatomical challenges in this cohort, rsRARP should not be dismissed and may offer distinct functional benefits in experienced hands.

## Supplementary Information

Below is the link to the electronic supplementary material.

Supplementary Material 1

Supplementary Material 2

## Data Availability

The data presented in this study are available on request from the corresponding author. The data are not publicly available due to ethical restrictions.
