# Supplementary material for: Comparison of Retzius-sparing versus anterior robotic-assisted radical prostatectomy in patients with prior transurethral resection of the prostate (TURP)
Source: World J Urol. 2025 Dec 5;44(1):23. doi: 10.1007/s00345-025-06112-3 (PMC12680673; doi:10.1007/s00345-025-06112-3)
Supplement: Supplementary file 1 — Supplementary Material 1 [file 345_2025_6112_MOESM1_ESM.docx]

**SUPPLEMENTARY MATERIAL**

**Supplementary Table 1 – Complication according to Clavien-Dindo up to 90 days postoperatively**

| *Parameter* | *Overall*  *(n=65)* | *rsRARP*  *(n=30)* | *aRARP*  *(n=35)* |
| --- | --- | --- | --- |
| Clavien Dindo postoperative complications classification |  |  |  |
| No complications | 55 (84.6) | 23 (76.7) | 32 (91.4) |
| Grade I | 4 (6.2) | 3 (10) | 1 (2.9) |
| Grade II | 0 (0) | 0 (0) | 0 (0) |
| Grade III  IIIa  IIIb | 2 (3.1)  4 (3.1) | 2 (6.7)  1 (3.3) | 0 (0)  1 (2.9) |
| Grade IV | 0 (0) | 0 (0) | 0 (0) |
| Grade V | 0 (0) | 0 (0) | 0 (0) |
| Missing | 2 (3.1) | 1 (3.3) | 1 (2.9) |
| rsRARP = Retzius sparing robotic assisted radical prostatectomy; aRARP = conventional robotic assisted radical prostatectomy  *Note: Some patients experienced more than one complication; all complications are reported individually and not limited to the most severe event.* | | | |
