# Supplementary material for: Comparison of Retzius-sparing versus anterior robotic-assisted radical prostatectomy in patients with prior transurethral resection of the prostate (TURP)
Source: World J Urol. 2025 Dec 5;44(1):23. doi: 10.1007/s00345-025-06112-3 (PMC12680673; doi:10.1007/s00345-025-06112-3)
Supplement: Supplementary file 2 — Supplementary Material 2 [file 345_2025_6112_MOESM2_ESM.docx]

**Figure legends**

Figure 1: Continence by grade over time and across surgery techniques

Figure 2: Kaplan-Meier curves with BCR-free survival according to surgical approach.
